# High-Dose Stereotactic Re-Irradiation of Recurrent High-Grade Gliomas: Clinical Outcome and Experience with AI-Based Target Volume Simulation

**DOI:** 10.3390/cancers17213423

**Published:** 2025-10-24

**Authors:** Anton Früh, Franziska Loebel, Bohdan Bodnar, Larissa Kilian, Martin Misch, Goda Kalinauskaite, Anne Kluge, Chiara Eitner, Julia Onken, Kerstin Rubarth, Daniel Zips, Peter Vajkoczy, Carolin Senger, Güliz Acker

**Affiliations:** 1 Department of Neurosurgery, Charité Universitaetsmedizin Berlin, 10117 Berlin, Germany; anton.frueh@charite.de (A.F.); martin.misch@charite.de (M.M.);; 2 Charité CyberKnife Center, Charité Universitaetsmedizin Berlin, 10117 Berlin, Germany; 3 Department of Radiation Oncology, Charité-Universitätsmedizin Berlin, 10117 Berlin, Germany; 4 Institute of Biometry and Clinical Epidemiology, Charité-Universitätsmedizin Berlin, 10117 Berlin, Germany

**Keywords:** radiosurgery, high-grade glioma, glioblastoma, radionecrosis, 18-FET-PET, AI-based tumor segmentation

## Abstract

**Simple Summary:**

High-grade gliomas almost inevitably recur after standard therapy, leaving limited treatment options. We evaluated stereotactic radiosurgery as a targeted re-irradiation strategy for small tumor recurrences, focusing on survival, local control, and treatment-related tissue damage. Our results show that this approach can provide meaningful disease control with acceptable safety, although radiation necrosis was frequent, particularly in tumors near the ventricular system. We also explored the role of artificial intelligence for tumor segmentation, which proved useful but still requires expert validation and advanced imaging. These findings support stereotactic radiosurgery as a feasible salvage treatment and highlight the need for improved strategies to reduce side effects and refine target definition.

**Abstract:**

Background/Objectives: Despite multimodal therapeutic concepts, treatment of recurrent malignant gliomas remains challenging. Stereotactic radiosurgery (SRS) may be a possible safe and effective non-invasive salvage treatment. In this study, we aim to investigate the SRS treatment outcomes using partly 18F-Fluorethylthyrosine (FET)-PET-imaging sequences for SRS treatment planning focusing on overall survival, event-free survival, and the incidence and factors influencing radiation necrosis (RN) occurrence. Additionally, we evaluated the potential application of AI-based tumor segmentation. Methods: We conducted a retrospective analysis of patients with recurrent malignant glioma treated with single-fraction or hypofractionated SRS at our institution. The outcomes assessed included local control, overall survival (OS), and local event-free survival (LEFS, defined as the interval until tumor recurrence or the onset of RN). We also performed a simulation analysis to assess the potential of AI-based tumor segmentation. Results: The study included 27 patients with a median age of 57 years and 41 lesions. The median OS post-SRS was 9.6 months and an LEFS of 5.2 months. Factors positively influencing OS and LEFS included the gross tumor volume (GTV) of the lesions before SRS therapy, presence of an IDH mutation, and lomustine treatment post-SRS. The incidence of RN post-SRS was 31.7%. RN was confirmed histopathologically in 15.4%, based on MRI in 46.2% and by FET-PET in 38.5% of lesions. In a simulation analysis, AI-based tumor segmentation reliably delineated all lesions, requiring only minimal manual adjustments to define target volumes. Conclusions: High-dose SRS is a feasible salvage treatment for small-volume recurrent high-grade gliomas, achieving local control and survival outcomes comparable to other re-irradiation strategies. IDH mutation, smaller tumor volume, and lomustine therapy were associated with improved survival. RN occurred frequently, particularly in periventricular lesions. AI-based tumor segmentation showed promise in well-defined satellite recurrences, but remains limited in cavity-adjacent lesions, underlining the need for expert review and ^18^FET-PET imaging.

## 1. Introduction

High-grade gliomas (HGGs) are among the most aggressive adult brain tumors, with an incidence of 3.2 per 100,000 people [1]. Despite multimodal treatment—resection, chemoradiation, and agents like temozolomide (TMZ) or lomustine [2,3]—recurrence within 6–9 months is common due to diffuse infiltration [4]. Management of recurrent HGG remains difficult, with limited treatment options and poor progression-free survival [5]. No consensus exists, though guidelines suggest re-resection, second-line chemotherapy, targeted therapy, or re-irradiation, all with modest efficacy and considerable side-effects [6]. As recurrences often arise near the prior radiation site, re-irradiation decisions hinge on timing, lesion size, proximity to organs at risk, age, Karnofsky Performance Status (KPS), and risk of radionecrosis (RN) [7,8]. Re-irradiation is generally recommended no earlier than 6 months after initial radiotherapy [9,10]. Stereotactic radiosurgery (SRS), delivered in single or hypofractionated sessions, offers a potential alternative for recurrent HGG, particularly for small lesions [11]. However, despite the technical advantages of SRS, the efficacy and safety of such treatment protocols in larger cohorts remain a topic of debate. Limited evidence and lack of large-scale studies continue to pose challenges in conclusively validating these approaches. Thereby, SRS known for its conformality, steep dose gradient, and precise targeting capabilities, may be particularly advantageous for administering radiation treatment to recurrent HGG located in close proximity to critical anatomical structures, such as the optic system or brainstem. This approach allows for re-irradiation while respecting the dose constraints of organs at risk (OAR), aiming to minimize radiation-induced damage to these vital areas [12,13]. While a small number of previous retrospective studies have indicated feasibility of robotic SRS [14,15,16,17,18,19], definitive reports on treatment outcome for selected patients with small inoperable recurrent HGG remain rare. Furthermore, the value of 18F-Fluorethylthyrosine (FET)-PET imaging for target volume determination is still controversial. Despite precise dose application, RN remains a common and severe side effect and factors influencing its occurrence are not yet fully understood. Furthermore, AI-based contouring emerges as a technical advantage for more accurate target volumes [20]. Therefore, the aim of the following study was to evaluate our institutional experience after SRS re-irradiation for recurrent HGG in terms of overall survival, event-free survival with a focus on incidence and factors influencing RN occurrence. In addition, also evaluate the potential of an AI-based tumor segmentation for future SRS planning.

## 2. Materials and Methods

### 2.1. Study Design and Patient Cohort

This retrospective single-center study was approved by the local ethics committee (EA 1/289/19) and conducted in accordance with the Declaration of Helsinki. All patients with recurrent WHO grade III or IV gliomas treated with single- or hypofractionated SRS using the CyberKnife-VSI-System (Accuray, Madison, WI, USA) between 2011 and 2021 were included. No exclusion criteria were applied.

### 2.2. SRS Treatment

Patients with small-volume recurrence ≥6 months after standard therapy (surgery/biopsy coupled with a course of radiotherapy and chemotherapy) were evaluated for salvage SRS by an interdisciplinary neuro-oncological board. Small-volume recurrence was defined by a GTV < 12 cm^3^, in line with clinical decision-making thresholds applied at our institution for SRS eligibility. Resolutions to proceed with re-irradiation utilizing robotic SRS were ultimately ratified by the interdisciplinary board following comprehensive evaluations of the individual cases. Treatment parameters, including dose and fractionation, were tailored based on prior radiation, lesion location, and OAR constraints per contemporary guidelines.

Planning computed tomography (CT, 0.75 mm, contrast-enhanced) was co-registered with MRI (contrast-enhanced T1-weighted 3D gradient-echo and fat-suppressed sequences). In selected cases, additional (^18^F-FET)-PET/MRI was fused for target definition. In our study, we used a tumor-to-brain ratio (TBR_max ≥ 2.0) as the reference threshold for defining metabolically active tumor regions. Although the PET-RANO expert panel later recommended a standardized lower cutoff (TBR_max ≥ 1.6) for identifying PET-positive tumor tissue, we maintained the institutional threshold of 2.0 for consistency within the retrospective dataset [21]. For target volume delineation, the attenuation-corrected FET-PET sequence and the contrast-enhanced T1-weighted MPRAGE MRI were fused with the planning CT. Only manual contouring was applied; no semi-automated PET-based biological tumor volume segmentation was used, as such approaches may be confounded by post-surgical changes and nonspecific tracer uptake [22]. FET-PET information served as a supportive modality alongside MRI, and the same TBR threshold (≥2.0) was consistently applied for both planning and follow-up evaluation to differentiate recurrence from radionecrosis.

**Figure 1:**
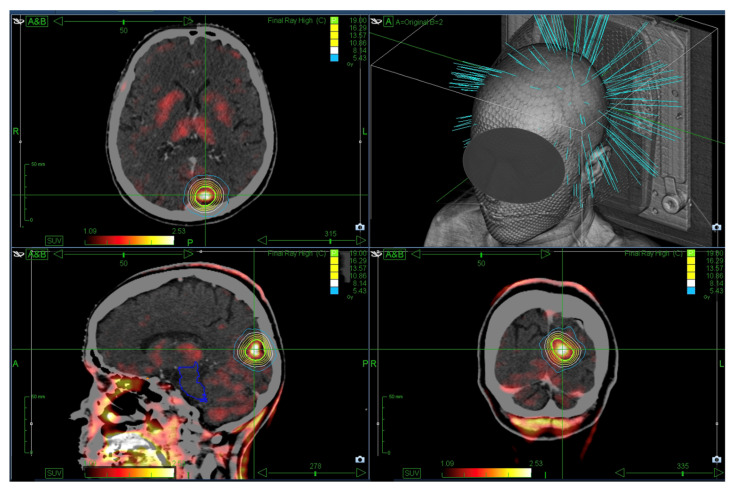
Exemplary single-session SRS plan showing a prescription dose of 1 × 19 Gy (green) enclosing the PTV. The planning CT was fused with FET-PET for target definition.

The gross tumor volume (GTV) was delineated using the most recent imaging, with a 0–2 mm margin to generate the planning target volume (PTV). Until June 2019, treatment planning was conducted using the MultiPlan (Accuray, Madison, WI, USA, versions 4.6.0 and 4.6.1) and subsequently with the Precision system (Accuray, Madison, WI, USA, versions 2.0.0.1, 2.0.1.1 and 3.2.0.0). Figure 1 displays an exemplary SRS treatment plan.

All patients received 4 mg dexamethasone post-SRS, with additional doses in the case of treatment-related edema or symptoms of raised intracranial pressure.

### 2.3. Data Collection

Patient data including age, gender, Karnofsky Performance Status (KPS), tumor volume, laterality and location, extent of resection (gross total [GTR], subtotal [STR], or biopsy), histopathological and molecular markers (WHO grade, IDH mutation, MGMT promoter methylation, ATRX loss, TP53 status), prior treatments, radiographic response, and treatment-related toxicity were retrospectively reviewed. WHO classification valid at the time of treatment was applied. SRS treatment parameters (prescription dose, isodose, planning target volume [PTV], treatment time) were also recorded. Follow-up included clinical evaluation and MRI every three months. Recurrence location was categorized as (i) within, (ii) marginal to, or (iii) outside the previously irradiated field. Marginal recurrence was defined as tumor regrowth at the periphery of the prior field where dose gradients were lower. Tumor response was classified according to RANO criteria: complete response (CR), partial response (PR), stable disease (SD), progressive disease (PD), and radiation necrosis (RN). RN was diagnosed via MRI (new or increased contrast enhancement within the radiation field) and confirmed with (FET)-PET (maximum tumor-to-brain-uptake-ratio < 2) or histology, where available. RN was categorized as definitive (dRN, PET- or histology-confirmed) or MRI-based (mRN). Periventricular lesions were defined by contact with the ventricular system. Overall survival (OS) was defined from initial diagnosis to death or last follow-up; local event-free survival (LEFS) as time to recurrence or RN. Adverse events were graded using Common Terminology Criteria for Adverse Events (CTCAE, Version 5).

### 2.4. Simulation of AI-Based GTV Segmentation

To evaluate the potential of AI in SRS planning, GTV_AIs were generated using RT Elements 4.5 (Brainlab AG, Munich, Germany, version 4.5.0.273) for cases with suitable MRI, including 21/26 (80.8%) satellite- and 14/15 (93.3%) resection-cavity-adjacent lesions (SL, RCL), and then reviewed by a radiosurgeon. In case of revision, corrected GTVs (GTV_AIc) were generated as reference and compared to GTV_AIs for volume overlap, potentially left out, and excess target volume in the simulation. The AI-based autosegmentation was performed exclusively on contrast-enhanced MRI data; FET-PET information was not included in the AI workflow. Manual contours used for comparison were also generated on MRI without the integration of PET data. Thus, the evaluation represents a direct comparison between MRI-based AI segmentations and MRI-based manual delineations. The AI-based Cranial Tumor Segmentation from RT Elements 4.5 (Brainlab AG, Munich, Germany, version 4.5.0.273) used in this study is a vendor-trained, commercially available model. No local retraining or fine-tuning was performed.

### 2.5. Statistical Analysis

Statistical analyses were performed using SPSS (v25.0), RStudio (v2023.06.0), and GraphPad Prism (v10). Data are presented as medians with interquartile ranges [IQRs] due to non-normal distribution. Statistical significance was defined as *p* ≤ 0.05. Given the exploratory nature of the study and limited sample size, no corrections for multiple testing were applied. Kaplan–Meier analysis with log-rank tests was used for survival comparisons. Univariate Cox regression was conducted using R packages survival, gtsummary, and survminer. Wilcoxon signed-rank tests were also applied where appropriate. For comparison of GTVs in simulation, we used descriptive volume comparisons and Dice-Coefficient.

## 3. Results

### 3.1. Baseline and SRS Treatment Characteristics

We included 27 patients (41 lesions) with a median age of 57 years [IQR 49.5–65] and 40.7% female. Epileptic seizures were the most common symptoms (41%). Median KPS at baseline was 80 [IQR 60–100] (see Table 1). FET-PET/MRI was performed in 13 of 27 patients (48.1%) prior re-irradiation and served as a complementary imaging modality.

WHO grade 3 tumors accounted for 20%, grade 4 for 80%. Most recurrences (68%) occurred within the previous radiation field, and 24% in marginal areas (see Table 2).

SRS was delivered in single fraction (75.6%) or hypofractionated regimens (24.4%), with doses ranging from 14 to 27 Gy. The median GTV was 0.78 cm^3^ [IQR 0.45–3.0] and the median PTV was 1.48 cm^3^ [IQR 1.0–5.4] (see Table 3).

### 3.2. Local Tumor Control and Clinical Outcome

**Figure 2:**
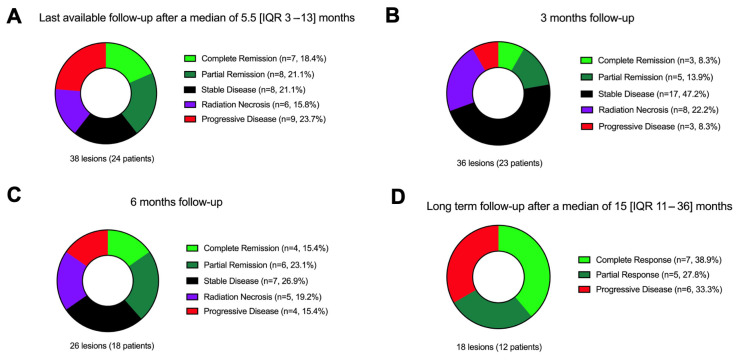
Local tumor control after SRS therapy divided into the following: (**A**) Last available follow-up after a median of 5.5 months. (**B**) A 3-month follow-up. (**C**) A 6-month follow-up. (**D**) Long-term follow-up after a median of 15 months. Abbreviations: CR = complete response, PR = partial remission, SD = stable disease, RN = radiation necrosis (both, definite and MRI-based), PD = progressive disease.

Among 36 evaluable lesions, 8.3% showed complete response, 13.9% partial response, 47.2% stable disease, 8.3% progression, and 22.2% RN (see Figure 2 and Supplement Figures S1 and S2) at a 3-month follow-up. Clinical follow-up was available for 24 patients (median follow-up duration 5.5 [IQR 3–13] months). KPS declined from a median of 80 to 70 (*p* = 0.005), 52% remained stable or improved, and 48% declined. Complications occurred in six patients (22.2%), and five CTCAE grade 1 and one grade 2 vestibular disorder (not persistent) was observed.

### 3.3. Radiation Necrosis (RN)

**Figure 3:**
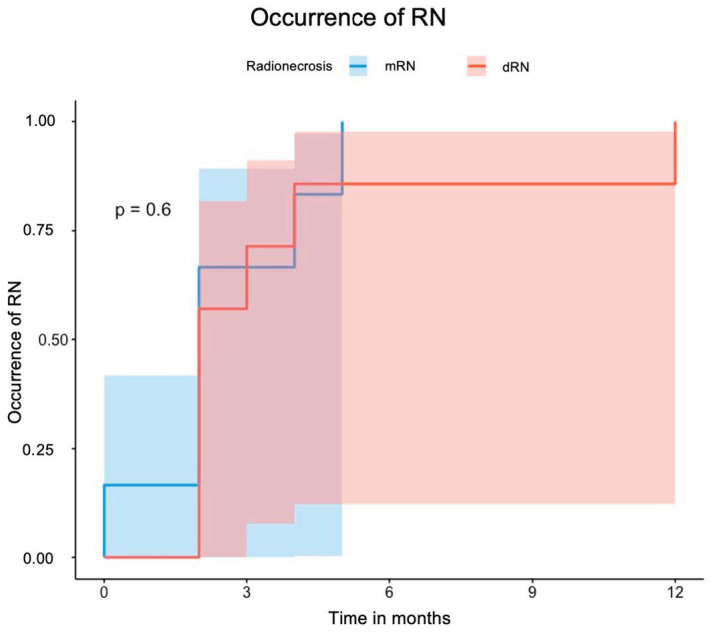
Curve for the occurrence of RN stratified according to mRN and dRN. Abbreviations: RN = radiation necrosis, dRN = definitive RN (PET- or histology-confirmed), mRN = MRI-based RN.

RN was diagnosed in 13 lesions (31.7%) among 11 patients. MRI confirmed six cases (mRN), FET-PET confirmed five, and histopathology confirmed two (dRN total: 7). RN occurred after a median of 2 months [IQR 2–4] post-SRS and was symptomatic in nine cases (69.2%). Symptoms were mostly managed with corticosteroids; two patients required surgery. Of seven post-SRS resections, five confirmed tumor recurrence and two RN. Five patients received bevacizumab. Periventricular location was the only independent predictor of RN (HR 6.13); 7 of 11 periventricular lesions (63.6%) developed RN. Figure 3 illustrates the occurrence of dRN and mRN. Uni- and multivariate analyses are provided in Table 4. Analysis limited to dRN yielded similar results.

### 3.4. Overall and Local Event-Free-Survival

**Figure 4:**
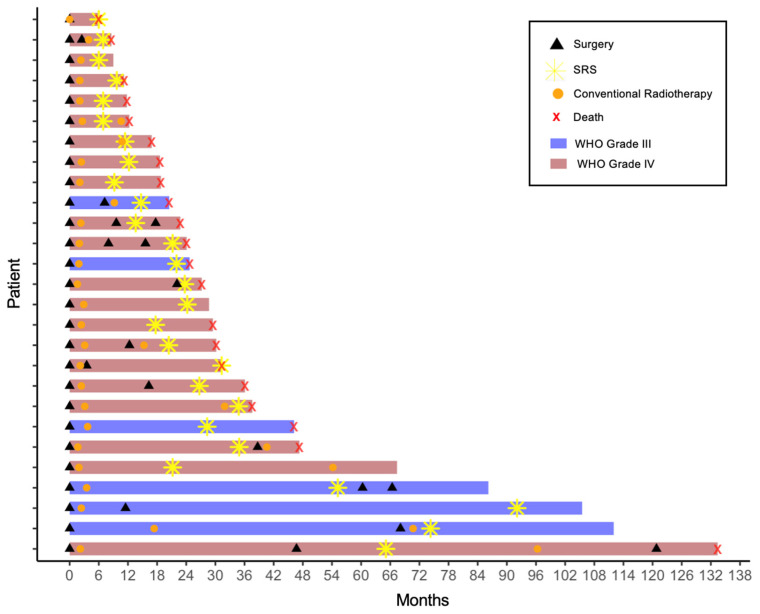
Swimmer plot illustrating individual patient timelines from first diagnosis to last follow-up or death. Each bar represents a patient, with segments indicating the timing of each intervention (surgery, conventional radiotherapy, stereotactic radiosurgery). Therapeutic modalities such as chemotherapy were omitted for clarity. In all cases the recurrence diagnosis was made immediately prior to the subsequent therapy. Abbreviations: SRS = stereotactic radiosurgery.

Following SRS, further therapies included re-resection (19.5%), re-irradiation (14.6%), chemotherapy (temozolomide, lomustine ± procarbazine, or bevacizumab), and TTFields (one patient). Figure 4 presents the individual courses from first diagnosis to last follow-up or death as a Swimmer Plot.

**Figure 5:**
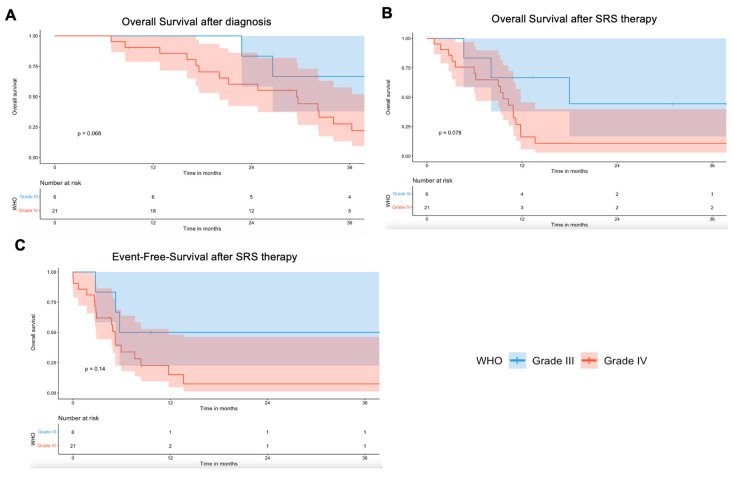
Kaplan–Meier survival curves for (**A**). Overall survival after initial diagnosis, (**B**) overall survival after SRS therapy, and (**C**) EFS after SRS therapy (censored data).

Median overall survival (OS) was 29.5 months [IQR 17.6–46.3]; post-SRS OS was 9.6 months [IQR 4.5–13.4]. Twenty-one patients (77.8%) died during follow-up. Due to limited distinction between pseudoprogression, RN, and tumor progression, all were considered events in calculating local EFS, with a median of 5.2 months [IQR 2.8–8.5]. Kaplan–Meier curves stratified by WHO grade are shown in Figure 5.

Univariate analysis identified the GTV (*p* = 0.01), IDH mutation (*p* = 0.01), and lomustine post-SRS (*p* = 0.02) as associated with survival. RN (dRN and mRN) had no significant impact on OS (see Table 5). Analysis restricted to dRN yielded similar results.

### 3.5. AI-Based GTV Segmentation

**Figure 6:**
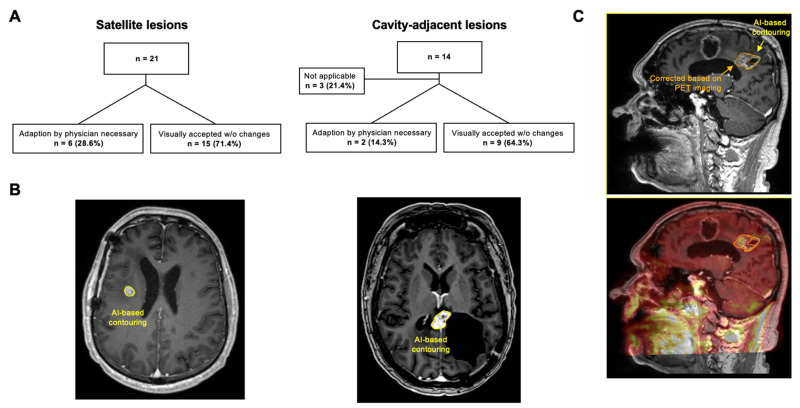
AI-based GTV segmentation. (**A**) Overview and results of AI-based segmentation, stratified by satellite lesions and cavity-adjacent lesions. (**B**) Two examples without the need for manual modifications: MRI imaging of a satellite lesion on the left and a cavity-adjacent lesion on the right. (**C**) Exemplary case, where AI was deemed not applicable, since the target could not be defined by contrast enhancement alone. Contrast-enhanced T1-weighted MRI with corresponding PET imaging below, showing the AI-generated contour (yellow) and manual PET-guided segmentation (orange). This case underscores the need for additional clinical or imaging data—such as sequential MRIs or PET—to accurately delineate targets near the resection cavity.

The AI correctly identified all 35 target lesions. In the visual evaluation, 3 of 14 (21.4%) RCLs were excluded from further analysis, as accurate AI-based segmentation was not feasible without additional information such as MRI follow-up or FET-PET (see Figure 6). In 6 out of 21 SLs, minor manual adjustments to GTV_AI were made by the radiosurgeon (Dice-Coefficient 0.64 (range: 0.4–0.76)). Importantly, 5 out of 6 corrected lesions were close to the ventricle. The median volume of SLs was 0.41 cm^3^ (range: 0.11–1.19 cm^3^) with a median GTV overlap of 69.7% (range: 28.6–94.1%). Potentially missed or excess volumes accounted for 30.3% (range: 5.9–71.4%) and 7.5% (range: 5.9–9.1%), respectively. For RCLs, minor corrections were needed in 2 of 11 cases, with a median Dice-Coefficient of 0.76. Due to the low number of corrections, no further comparison was performed. To further evaluate segmentation performance, we performed a subgroup comparison between lesions in which the AI correctly delineated the target and those in which segmentation failed or required correction. AI-based segmentation performed particularly well for satellite lesions (SLs) with distinct contrast enhancement and clear margins. Manual corrections were required in six cases, five of which were located near the ventricular system, where the complex anatomy and proximity to cerebrospinal fluid spaces hindered accurate contour generation. For RCLs, corrections were necessary in two cases, mainly due to scar tissue-related contrast enhancement that complicated delineation.

## 4. Discussion

The present data suggest that high-dose SRS is a feasible salvage treatment for small-volume recurrences of HGG offering local control and survival benefits with manageable toxicity. Periventricular location emerged as an independent predictor of RN. AI-based segmentation holds high potential for the SRS planning.

Radiosurgery has been used in case series as a salvage treatment in recurrent high-grade glioma after initial concurrent chemoradiation and other therapies. These initial studies and meta-analysis have shown feasibility concerning a safe treatment and adequate local tumor control but a high risk of development of RN [14,19,23]. Our results are in line with previous studies and show that SRS may a feasible strategy for locally confined recurrence of high-grade gliomas with adequate local control, but a high risk of development of RN.

In the present study nearly half of the patients underwent pretherapeutic FET-PET-MRI imaging (48.1%). The basic procedure of FET-PET SRS planning is comparable to an established protocol used for meningiomas [24]. Importantly, the very recent prospective multicenter randomized trial (NOA 10/ARO 2013-1) analyzed the role of PET in irradiation of recurrent glioblastoma without verifying any advantage of FET-PET-based planning [25,26]. However, here a total of 39 Gy á 3 Gy per day was applied. The role of FET-PET-based planning may be more prominent in radiosurgery where less margins are applied. This should be further analyzed. In our series, FET-PET was primarily used as an adjunct to MRI for target definition in selected cases, particularly when contrast enhancement on MRI was ambiguous or when surgical changes complicated anatomical delineation. Although FET-PET information did not systematically alter the final GTV in our retrospective cohort, it frequently provided additional metabolic information that supported interdisciplinary tumor-board decisions. Moreover, FET-PET proved useful during follow-up, helping to differentiate radionecrosis from true progression in ambiguous cases. While our study was not designed to quantitatively assess the impact of FET-PET on planning or outcome, these findings underscore its supportive role in radiosurgical workflow and the need for standardized integration in future prospective studies.

AI-based advances are increasingly shaping radiotherapy. In our pilot cohort, AI autosegmentation detected all lesions; 71.4% of SLs and 64.3% of RCLs were visually accepted without modification. In cases requiring minor adjustments, differences in volume and the Dice score should be interpreted with caution due to the small lesion sizes. Accuracy was especially limited near ventricles, indicating room for improvement. Importantly, 21.4% of resection cavity-adjacent lesions required additional imaging, such as FET-PET, underscoring the value of combining AI with multimodal imaging and the continued role of clinical expertise. Overall, current AI tools may improve planning efficiency and support more standardized target delineation in clinical practice.

Only 8.3% of lesions showed progression at three months. Median OS was 29.5 [IQR 17.6–46.3] months overall and 9.6 [IQR 4.5–13.4] months post-SRS, indicating that SRS offers a viable salvage option alongside other re-irradiation strategies such as 10 × 3.5 Gy or 13 × 3 Gy. IDH mutation, smaller GTVs, and lomustine therapy were associated with improved survival. The ongoing LEGATO phase III trial (NCT05904119) will further clarify the role of lomustine with or without re-irradiation in recurrent glioblastoma [27].

The survival outcomes in our study align with previous reports. In a large cohort of 128 patients with 161 recurrent HGG lesions, median survival from initial diagnosis was 32 months and 11.5 months after SRS [15]. Guan et al. reported a median OS of 17.6 months following hypofractionated SRS [18]. Even if the survival benefit is modest, single-fraction or hypofractionated therapy offers improved quality of life compared to prolonged treatment regimens, especially in patients with limited prognosis. Lévy et al. [28] found a median survival of 14 months and relapse-free survival of 3.7 months after SRS in 13 patients with recurrent malignant gliomas. Prolonged PFS was associated with patient age, total dose, dose per fraction, and number of fractions. Improved OS after radiosurgery was linked to age < 40 years, prior salvage surgery, and additional post-SRS therapies. Other studies identified performance status at re-irradiation (Karnofsky scale) as a key predictor of PFS. Median OS and PFS after SRS for recurrent malignant gliomas were reported as 9 and 3 months, respectively [29]. A further comprehensive report of the literature regarding SRS for the treatment of HGG can be found in Supplementary Table S1 [5,14,16,17,18,19,23,28,30,31,32,33,34,35,36,37,38,39,40,41,42,43,44,45,46,47,48,49,50,51,52,53,54,55,56,57,58,59,60,61,62,63,64,65,66,67,68,69,70,71,72,73,74,75,76,77,78,79,80,81,82].

Despite potential survival benefits, RN remains a relevant complication after SRS for recurrent HGG. Differentiating RN from true progression is clinically challenging but essential for management and prognosis [83]. RN occurred in 31.7% of lesions in our cohort, which is higher than in previous reports [72,83]. This may be attributable to the high radiation dose, the frequent infield re-irradiation, and the inclusion of MRI-based RN without histological or PET confirmation. The underlying mechanisms are multifactorial and include vascular injury, inflammation, and blood–brain barrier disruption [83]. RN was symptomatic in 69.2% of cases in our cohort. In an early study, RN occurred in 4 of 61 glioblastoma patients treated with three to six fractions [72]. In a larger cohort of 128 recurrent HGG patients with 161 lesions, the incidence was ~6%, with no high-grade toxicity reported [15]. Similar RN rates (11.4%) were found elsewhere [29]. In the recent prospective “GLIAA-Trial” [25], RN rates reached up to 25%—more closely aligning with our findings. Our higher incidence may reflect the high cumulative dose in infield re-irradiation and inclusion of MRI-based RN. While total doses >40 Gy have been linked to RN, hypofractionated SRS has been proposed as a safer alternative [10,18,84,85]. Importantly, RN or pseudoprogression does not necessarily indicate poor prognosis; some studies report longer OS in patients with early post-SRS imaging changes [47]. In our cohort, periventricular tumor location significantly increased RN risk. Few prior studies have addressed this association [86,87], but it may relate to the radiosensitivity of neural stem cells in the subependymal zone [88,89]. Further investigation in larger, prospective cohorts is warranted.

This study is limited by its retrospective design, small sample size, and heterogeneous cohort. Diagnostic uncertainty regarding RN versus true progression remains, despite MRI and FET-PET imaging, and no MR spectroscopy was performed. It should be emphasized that the very low hazard ratio for IDH mutation observed in the multivariate Cox regression (HR = 0.07) must be interpreted with caution due to the small number of IDH-mutant cases (n = 8) in our cohort. While this finding is consistent with the well-established prognostic benefit of IDH mutation, the limited sample size increases the risk of effect overestimation. The inclusion of only one IDH-mutant glioblastoma precludes subgroup analysis and limits interpretation under the current WHO classification. Nonetheless, the data reflect real-world outcomes of SRS in recurrent glioma. Given recent evidence supporting hypofractionated SRS [90], prospective studies are warranted to further define its role.

Patients in the post-irradiation setting can develop both radionecrosis and disease progression over time. Due to the study design, it is challenging to fully account for this dynamic process. Furthermore, it should be acknowledged that the AI-based Cranial Tumor Segmentation from RT Elements 4.5 (Brainlab AG, Munich, Germany, version 4.5.0.273) used in this study was not specifically trained on recurrent glioma cases. This limitation may have affected the accuracy of AI-based delineation, particularly in resection cavity-adjacent lesions where MRI alone often provides insufficient contrast and FET-PET can provide valuable complementary information.

## 5. Conclusions

High-dose SRS combined with multimodal technique like PET imaging and AI for precise target delineation appears a feasible salvage option for small-volume recurrent HGG, offering local control and survival outcomes comparable to other re-irradiation strategies. The high incidence of radionecrosis, particularly in periventricular lesions, highlights the need for further research into risk stratification and optimal treatment protocols.

## Figures and Tables

**Table 1:** Baseline characteristics of included patients.

	Patients (n = 27)
Age, Median (IQR)	57 (49.5–65)
Gender, n (%)	
Female	11 (40.7)
Male	16 (59.3)
Leading Symptom, n (%)	
Epilepsy of any kind	11 (41)
Frontal deficiencies	2 (7.4)
Headache	2 (7.4)
Motoric deficiencies	1 (3.7)
Sensory deficiencies	2 (7.4)
Speech deficiencies	3 (11)
Other	6 (22)
Prior Surgeries, n (%)	
1	14 (51.9)
2	10 (37.0)
3	3 (11.1)
KPS Score (%), n (%)	
60	3 (11.1)
70	6 (22.2)
80	5 (18.5)
90	9 (33.3)
100	4 (14.8)
Amount of lesions, n (%)	
1	17 (63.0)
2	6 (22.2)
3	4 (14.8)

Abbreviations: KPS: Karnofsky Performance Status; IQR: interquartile range; n, number.

**Table 2:** Characteristic of treated lesions.

		Lesions (n = 41)
Diagnosis (WHO 2016), n (%)		
Anaplastic Astrocytoma		4 (9.8)
Anaplastic Xanthoastrocytoma		2 (4.9)
Anaplastic Oligoastrocytoma		2 (4.9)
Glioblastoma		33 (80)
WHO grade, n (%)		
III		8 (19.5)
IV		33 (80.5)
Localization, n (%)		
Basal ganglia		1 (2.4)
Corpus callosum		2 (4.9)
Frontal		15 (37)
Frontoparietal		1 (2.4)
Frontotemporal		1 (2.4)
Hippocampal		2 (4.9)
Lateral ventricle		3 (7.3)
Occipital		5 (12)
Parietal		3 (7.3)
Parietooccipital		1 (2.4)
Precentral		1 (2.4)
Temporal		6 (15)
Periventricular location, n (%)		11 (26.8)
Located within previous irradiated field, n (%)		
Marginal area		10 (24)
No		3 (7.3)
Yes		28 (68
Status		
	WHO grade III lesions (n = 33)	All lesions (n = 41)
IDH mutated, n (%)	3 (9.1)	8 (19.5)
MGMT methylated, n (%)	20 (60.6)	21 (55.3)
Unknown	0	3
TP53 mutation, n (%)	9 (40.1)	13 (39)
Unknown	7	8
ATRX loss, n (%)	3 (9.1)	5 (14.7)
Unknown	5	7

Abbreviations: IQR: interquartile range; n, number. Note: “Oligoastrocytoma” has been retained here only to reflect historical diagnoses valid at the time of treatment (WHO 2016).

**Table 3:** Treatment characteristics.

	Lesions (n = 41)
**Treatment prior SRS therapy**	
Number of surgeries prior to SRS, n (%)	1: 23 (56.1); 2: 13 (31.7); 3: 5 (12.2)
Extent of resection of first surgery (n = 41), n (%)	Biopsy: 2 (4.9); STR: 21 (51.2); GTR: 18 (43.9)
Extent of resection of second surgery (n = 18), n (%)	STR: 12 (29.3); GTR: 6 (14.6)
Extent of resection of third surgery (n = 5), n (%)	STR: 1 (2.4); GTR: 4 (9.8)
Time from last surgery to SRS, months, median [IQR]	12 [7–24]
Number of radiations of the patient prior to SRS, n (%)	1: 35 (87.8); 2: 5 (12.2)
Dose of first radiation, Gy, median [IQR]	60 [40.5–66]
Dose of second radiation, Gy, median [IQR]	50.4 [49.4–60]
Time from last radiation to SRS, months, median [IQR]	12 [5–24]
Other therapies prior to SRS, n (%)	
TMZ	41 (100)
Metronomic TMZ	13 (31.7)
Lomustine	9 (22.0)
Bevacizumab	0 (0.0)
NovoTFF	7 (16.0)
**SRS treatment**	
**Single Session SRS, n (%)**	31 (75.6)
Prescription dose, Gy, median [IQR]	21 [20–21]
Isodose line, %, median [IQR]	70 [70]
Mean dose, Gy, median [IQR]	25.4 [24.3–25.8]
Minimum dose, Gy, median [IQR]	19.6 [18.9–20.4]
Maximum dose, Gy, median [IQR]	30.0 [28.6–30.0]
Gross tumor volume, cm^3^, median [IQR]	0.68 [0.39–1.26]
Planning target volume, cm^3^, median [IQR]	1.19 [0.92–1.70]
Coverage, %, median [IQR]	99.2 [98.6–99.7]
**Hypofractionation, n (%)**	10 (24.4)
**3 Fractions, n (%)**	9 (22.0)
Prescription dose, Gy, median [IQR]	24 [24–27]
Mean dose, Gy, median [IQR]	29.6 [29.2–32.8]
Minimum dose, Gy, median [IQR]	23.1 [22.2–26.1]
Maximum dose, Gy, median [IQR]	34.3 [34.3–38.6]
Gross tumor volume, cm^3^, median [IQR]	4.48 [3.16–7.10]
Planning target volume, cm^3^, median [IQR]	7.40 [5.77–11.00]
Coverage, %, median [IQR]	99.0 [98.3–99.8]
**5 Fractions, n (%)**	1 (2.4)
Prescription dose, Gy	25
Isodose line, %	70
Mean dose, Gy	29.5
Minimum dose, Gy	16.8
Maximum dose, Gy	35.7
Gross tumor volume, cm^3^	4.01
Planning target volume, cm^3^	19.67
Coverage, %	99.9
**Chemotherapy during SRS, n (%)**	
No additional chemotherapy	29 (70.7)
Metronomic TMZ	8 (29.6)
Lomustine	1 (2.4)
Abemaciclib	1 (2.4)
**Treatment post SRS therapy, n (%)**	
Re-Surgery	8 (19.5)
Re-Irradiation	6 (14.6)
TMZ	5 (12.2)
Metronomic TMZ	17 (41.5)
PC	5 (12.2)
Lomustine	12 (29.3)
Bevacizumab	5 (12.2)
NovoTFF	1 (2.4)

Abbreviations: SRS: stereotactic radiosurgery; IQR: interquartile range; Gy: gray; n, GTR: gross total resection, number, TMZ: temozolomide, TTF: tumor treating fields, PC: procarbazine and lomustine, STR: subtotal resection. Bold and underlined entries represent subheadings in the table for clarity.

**Table 4:** Risk factors for the occurrence of radionecrosis (RN).

	HR (95% CI)	*p*-Value
Risk for the occurrence of RN (univariate)		
Male gender	0.57 (0.14–2.21)	0.40
Age	0.95 (0.89–1.02)	0.20
WHO grade IV	0.83 (0.17–4.72)	0.80
IDH mutated	0.58 (0.08–3.02)	0.50
MGMT methylated	2.07 (0.49–9.72)	0.30
TP53	0.86 (0.18–3.87)	0.80
Periventricular location	6.13 (1.40–31.1)	0.02
Time from last RT to SRS	0.98 (0.94–1.01)	0.30
PTV	0.98 (0.81–1.15)	0.80
GTV	1.01 (0.72–1.38)	>0.9
Risk for the occurrence of RN (multivariate)		
Age	0.95 (0.88–1.02)	0.15
Periventricular location	6.73 (1.44–38.5)	0.02

Abbreviations: CI = confidence interval; RT = radiation therapy; RN = radionecrosis (diagnosed based on MRI scans, ^18^F-FET-PET/MRI or histopathology), CK-SRS = CyberKnife stereotactic radiosurgery; HR = hazard ratio.

**Table 5:** Risk factors for poor (event-free-) survival (cox regression).

	HR (95% CI)	*p*-Value
**Risk for poor overall survival from diagnosis**		
**Univariate**		
Male gender	1.05 (0.41–2.65)	0.90
Age	1.03 (0.99–1.07)	0.13
WHO grade IV	3.02 (0.87–10.48)	0.05
GTV	1.27 (1.08–1.50)	0.01
IDH mutated	0.07 (0.01–0.57)	0.01
MGMT methylated	0.62 (0.25–1.50)	0.30
TP53	0.94 (0.32–2.76)	0.90
ATRX loss	0.17 (0.02–1.30)	0.09
Amount of lesions	0.66 (0.33–1.32)	0.20
No surgery after SRS	3.11 (0.71–13.7)	0.13
No radiation after SRS	2.91 (0.66–12.8)	0.20
No TMZ after SRS	2.41 (0.96–6.04)	0.06
No lomustine after SRS	4.19 (1.2–14.5)	0.02
Bevacizumab after SRS	083 (0.23–2.95)	0.80
Occurrence of RN *	0.55 (0.21–1.44)	0.20
Lesions located within previous irradiated field	0.66 (0.11–1.94)	0.50
**Multivariate**		
WHO grade IV	0.73 (0.17–3.32)	0.70
GTV	1.20 (1.01–1.42)	0.04
IDH mutated	0.09 (0.01–1.03)	0.05
No lomustine after SRS	3.32 (0.89–11.02)	0.08
**Risk for short event-free-survival after SRS**		
**Univariate**		
Male gender	1.69 (0.66–4.34)	0.30
Age	1.03 (1.0–1.07)	0.10
WHO grade IV	2.48 (0.72–8.50)	0.15
GTV	1.21 (1.03–1.44)	0.02
IDH mutated	0.09 (0.01–0.72)	0.02
MGMT methylated	0.76 (0.31–1.86)	0.50
TP53	0.51 (0.17–1.54)	0.20
ATRX loss	0.18 (0.02–1.42)	0.10
Amount of lesions	0.62 (0.31–1.23)	0.20
No surgery after SRS	4.04 (0.90–18.1)	0.07
No radiation after SRS	3.24 (0.71–14.7)	0.13
No TMZ after SRS	1.7 (0.67–4.29)	0.30
No lomustine after SRS	3.73 (1.07–13.0)	0.04
No bevacizumab after SRS	0.73 (0.21–2.52)	0.60
**Multivariate**		
GTV	1.20 (1.01–1.44)	0.03
IDH mutated	0.11 (0.01–0.93)	0.04
No lomustine after SRS	3.32 (0.93–11.0)	0.06

Abbreviations: CI = confidence interval; SRS = stereotactic radiosurgery; HR = hazard ratio; * using only definitive RNs (FET-PET and histological based) did not influence the results; GTV = gross total volume; tMZ: temozolomide. Bold and underlined entries represent subheadings in the table for clarity.

## Data Availability

The data presented in this study are available on reasonable request from the corresponding author.

## Supplementary Materials

The following supporting information can be downloaded at https://www.mdpi.com/article/10.3390/cancers17213423/s1; Figure S1: Sankey plot showing the flow of the local tumor control between the 3-month and long-term follow-up. Figure S2: Exemplary 67-year-old female patient. Table S1: Comprehensive report of the literature regarding SRS for the treatment of HGG.

## Abbreviations

The following abbreviations are used in this manuscript:

AI	Artificial Intelligence
CR	Complete Response
CTCAE	Common Terminology Criteria for Adverse Events
dRN	Definite Radiation Necrosis
mRN	MRI-based Radiation Necrosis
FET	Fluorethyltyrosine
GTR	Gross Total Resection
GTV	Gross Total Volume
HGG	High Grade Glioma
IQR	Interquartile Range
KPS	Karnofsky Performance Score
PR	Partial Remission
PTV	Planning Target Volume
RCL	Resection Cavity-Adjacent Lesion
RN	Radiation Necrosis
SD	Stable Disease
SL	Satellite Lesion
STR	Subtotal Resection
TMZ	Temozolomide
