# Supplementary material for: High-Dose Stereotactic Re-Irradiation of Recurrent High-Grade Gliomas: Clinical Outcome and Experience with AI-Based Target Volume Simulation
Source: Cancers (Basel). 2025 Oct 24;17(21):3423. doi: 10.3390/cancers17213423 (PMC12606762; doi:10.3390/cancers17213423)
Supplement: Supplementary file 1 [file cancers-17-03423-s001.zip › cancers-3886556-supplementary.pdf]

cancers

Article

High-Dose Stereotactic Re-Irradiation of Recurrent High-Grade Gliomas: Clinical Outcome and Experience with AI-Based Target Volume Simulation

Anton Früh [1,†], Franziska Loebel [1,2,†], Bohdan Bodnar [2], Larissa Kilian [2,3], Martin Misch [1], Goda Kalinauskaite [3], Anne Kluge [2,3], Chiara Eitner [2,3], Julia Onken [1], Kerstin Rubarth [4], Daniel Zips [3], Peter Vajkoczy [1], Carolin Senger [2,3,*,‡] and Güliz Acker [1,2,‡]

1 Department of Neurosurgery, Charité Universitaetsmedizin Berlin, 10117 Berlin, Germany; anton.frueh@charite.de (A.F.); martin.misch@charite.de (M.M.)
2 Charité CyberKnife Center, Charité Universitaetsmedizin Berlin, 10117 Berlin, Germany
3 Department of Radiation Oncology, Charité-Universitätsmedizin Berlin, 10117 Berlin, Germany
4 Institute of Biometry and Clinical Epidemiology, Charité-Universitätsmedizin Berlin, 10117 Berlin, Germany
* Correspondence: carolin.senger@charite.de; Tel.: +49-30-450-627698
† These authors contributed equally to this work.
‡ These authors also contributed equally to this work.

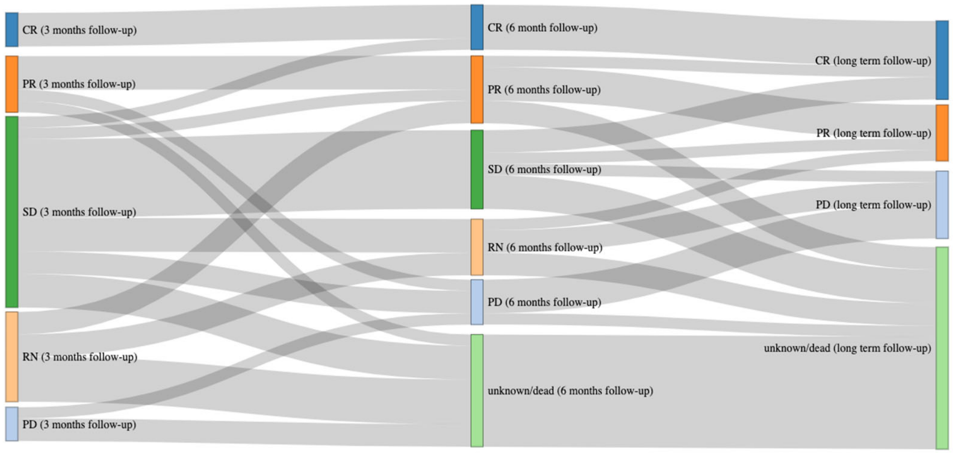

Figure S1. Sankey plot showing the flow of the local tumor control between the 3 months and long-term follow-up. Abbreviations: CR = Complete response, PR = Partial Remission, SD = Stable Disease, RN = Radiation Necrosis (both, definite and MRI-based), PD = Progressive Disease.

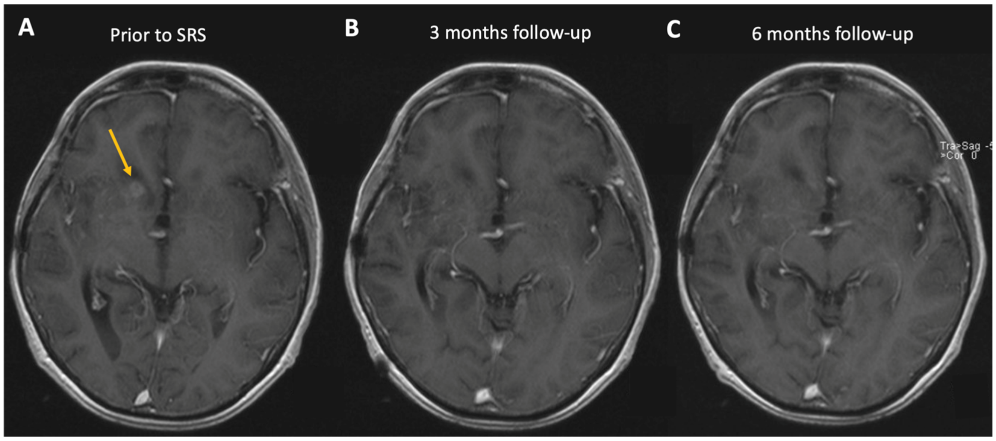

Figure S2. Exemplary 67 year-old female patient. She was initially suffering from a Glioblastoma (WHO grade IV, IDH wildtype, MGMT methylated) and received postoperative radiochemotherapy according to STUPP, followed by 6 cycles of TMZ and Tumor Treating Fields therapy. The patient was treated with a single session SRS (21 Gy, 70% Isodose) and showed a complete response after 3- and 6-months. At the 1-year follow-up, the patient also demonstrated clinical and radiographical stability. Axial T1-weighted contrast-enhanced MRI scans A. Prior SRS therapy B. Three months follow-up showing a complete response C. Six months follow-up showing a complete response.

.

Table S1. Comprehensive report of literature regarding SRS for the treatment of HGG

Title and year of publication	Authors and Journal	Study design	Number of patients	Endpoints/Analysis (Selection)	Results
Safety and efficacy of Hypofractionated stereotactic radiosurgery for high-grade Gliomas at first recurrence: a single-center experience - 2021	Guan, Y., Xiong, J., Pan, M. et al. - BMC Cancer 21	Retrospective data analysis	70 (49 Patients had an initial diagnosis of GBM, and the rest (21) were confirmed to be WHO grade 3 gliomas)	Investigate the efficacy and safety of hypofractionated stereotactic radiosurgery as a first-line salvage treatment for in-field recurrence of high-grade gliomas<br>primary endpoint: OS<br>secondary endpoints: PFS	-Median OS after salvage treatment: 17.6 months<br>-No grade 3 or higher toxicities was recorded<br>-Median PFS: 7 months
Randomized prospective trial of fractionated stereotactic radiosurgery with chemotherapy versus chemotherapy alone for bevacizumab-resistant high-grade glioma - 2020	Bergman D, Modh A, Schultz et al.- J Neurooncol.	Randomized prospective trial	35 (29 had glioblastoma (WHO IV) and 6 had anaplastic glioma (WHO III))	To compared FSRS plus BEV-based chemotherapy versus BEV-based chemotherapy alone for BEV-resistant recurrent malignant glioma<br>primary endpoint: PFS, local control<br>secondary endpoint: OS	-PFS in Patients treated only with BEV: 1.8 months<br>-PFS in Patients treated with FSRS: 5.1 months<br>-LC at 2 months in Patients treated with FSRS: 82%<br>-LC at 2 months in Patients treated only with BEV: 27%<br>-Median OS: 6.6 months (7.2 months with FSRS vs 4.8 months with chemotherapy alone)
Early versus delayed postoperative radiotherapy for treatment of low-grade gliomas - 2020	Dhawan S, Patil CG, Chen C, et al. - Cochrane Database Syst Rev.	Meta-Analysis	311	Assess the effects of early postoperative radiotherapy versus radiotherapy delayed until tumour progression for low-grade intracranial gliomas in people who had initial biopsy or surgical resection<br>primary endpoint: PFS<br>secondary endpoint: OS	- Median PFS: 5.3 years in the early radiotherapy group; 3.4 years in the delayed radiotherapy group<br>-Median OS: 7.4 years in the early radiotherapy group; 7.2 years in the delayed radiotherapy group
Survival after hypofractionation in glioblastoma: a systematic review and	Trone JC, Vallard A, Sotton S, et al. - Radiat Oncol.	Meta Analyse (11 studies)	1738	Analysed data from all non-comparative trials testing the impact of hypofractionation, radiosurgery and hypofractionated stereotactic RT in first line<br>primary endpoint: OS	-Median OS of the hypofractionated radiotherapy + chemotherapy arms:16.8 months<br>-Median OS of the hypofractionated

meta-analysis - 2020					radiotherapy arms: 8.9 months<br>-Median OS in trials based on non-stereotactic hypofractionation: 6.7 months<br>-Median OS of the hSRT arms: 12.7 months
External beam radiation dose escalation for high grade glioma - 2020	Khan L, Soliman H, Sahgal A, et al. - Cochrane Database Syst Rev.	Meta-Analysis	1537 (11 RCTs)	Assess the effects of postoperative external beam radiation dose escalation in adults with HGG<br>primary endpoint: OS	-Overall survival was similar between hypofractionated and conventional radiotherapy in five trials (943 participants)<br>-Overall survival was similar between hypofractionated and conventional radiotherapy in the subset of two trials (293 participants) which included participants aged 60 years and older
A phase I/II trial of 5-fraction stereotactic radiosurgery with 5-mm margins with concurrent temozolomide in newly diagnosed glioblastoma: primary outcomes - 2020	Azoulay M, Chang SD, Gibbs IC et al. - Neuro Oncol.	Phase I/II trial	30	Determine the maximum tolerated dose of 5-fraction SRS with 5-mm margins delivered with concurrent temozolomide in newly diagnosed GBM<br>primary endpoint: maximum tolerated dose<br>secondary endpoint: OS, PFS	-MTD for hypofractionated radiotherapy over 5 consecutive days with 5-mm margins in targets up to 150 cm3 with concurrent TMZ is 40 Gy in 5 fractions<br>-consider a lower dose level of 35 Gy in 5 fractions if combined with agents that may potentiate radiation toxicity<br>-OS: 14.8 months<br>-PFS: 8.2 months
Study Protocol: Early Stereotactic Gamma Knife Radiosurgery to Residual Tumor After Surgery of Newly Diagnosed Glioblastoma - 2019	Brehmer S, Grimm MA, Förster A, Seiz-Rosenhagen et al. - Neurosurgery	single center, open-label, prospective, single arm, phase II study	50	Assess safety and efficacy of adding SRS to the standard treatment of GBM in patients with postoperative residual tumor<br>primary endpoint: PFS	-PFS: 13.8 months
Clinical Efficacy of CyberKnife Radiosurgery	Zhang J, Liu Q, Yuan Z, et	Retrospective data analysis	21	Analyze and evaluate the clinical efficacy of CyberKnife for brainstem gliomas	-One year OS: 87.5%<br>-Two year OS: 52.4%<br>-Median OS: 19 months, with 5 patients still alive

for Adult Brainstem Glioma: 10 Years Experience at Tianjin CyberKnife Center and Review of the Literature - 2019	al. -Front Oncol.			primary endpoint: 1-/2-year OS	
Phase II study of hypofractionated radiation therapy in elderly patients with newly diagnosed glioblastoma with poor prognosis - 2019	Navarria P, Pessina F, Cozzi L, et al. - Tumori.	Prospective Phase 2 trial	30	Evaluate HFRT given at therapeutic effective doses in a phase II study.<br>primary endpoint: PFS<br>secondary endpoint: OS and incidence of toxicity	-Median PFS: 5 months<br>-Median OS: 8 months<br><br>-No increase in steroid drugs was required during radiotherapy treatment and a reduction was possible in 12
Phase I trial of alisertib with concurrent fractionated stereotactic re-irradiation for recurrent high grade gliomas - 2019	Song A, Andrews DW, Werner-Wasik M, et al.	Prospective Phase 1 trial	17	Analyze the efficacy of alisertib with concurrent fractionated stereotactic re-irradiation for recurrent high grade gliomas<br>primary endpoint: OS<br>secondary endpoint: PFS	-OS at 6 months: 88.2%<br>-Median ST: 11.1 months<br><br>-PFS at 6 months: 35.3%<br>-Median time to progression: 4.9 months
Efficacy and Safety of Hypofractionated Stereotactic Radiotherapy for Recurrent Malignant Gliomas: A Systematic Review and Meta-analysis - 2019	Hu YJ, Chen D, Zhang LF et al. - World Neurosurg.	Meta Analyse	861 patients (26 studies)	A descriptive analysis of the median overall survival and meta-analysis of the reported rates of RN<br>primary endpoint: OS	-Median OS ranged from 8.6 to 18 months
Phase I Trial of Radiosurgery Dose Escalation Plus Bevacizumab in Patients With Recurrent/ Progressive	Abbassy M, Missios S, Barnett GH et al. - Neurosurgery	Prospective Phase 1 trial	9	Determine the safety of dose escalation of single-fraction radiosurgery for rGBM in the setting of bevacizumab therapy<br>primary endpoint: Median OS<br>secondary endpoint: PFS	-Median OS: 13 months<br>-Median PFS: 7.5 months<br><br>-Pre-SRS bevacizumab treatment was associated with a reduction of the mean volume of the enhancing lesion from 4.7 to 2.86 cm3 on the day of SRS

Glioblastoma - 2018					
Salvage fractionated stereotactic re-irradiation (FSRT) for patients with recurrent high grade gliomas progressed after bevacizumab treatment – 2018	Shi W, Blomain ES, Siglin J et al. - J Neurooncol.	Retrospective data analysis	36	Evaluate the feasibility and efficacy of fractionated stereotactic re-irradiation for patients with high grade gliomas after Bevacizumab treatment<br>primary endpoint: Median OS<br>secondary endpoint: PFS	-OS from initial diagnosis: 24.9 months<br>-Median OS from FSRT: 4.8 months<br>-Median OS from initiation of bevacizumab: 13.4 months<br><br>-Median progression free survival after FSRT: 3.9 months
Long-Term Outcomes Following Conventionally Fractionated Stereotactic Boost for High-Grade Gliomas in Close Proximity to Critical Organs at Risk – 2018	Repka MC, Lei S, Campbell L. et al. - Front Oncol.	Retrospective data analysis	30	Showing long-term outcomes in patients treated with conventionally fractionated stereotactic boost for tumors in close proximity to critical structures<br>primary endpoint: Median OS<br>secondary endpoint: Tumor Progression	-Median OS: 45 months<br>-5YOS: 32.5%<br>-Median freedom from local progression: 45 months<br>-5-year freedom from local progression: 29.7%
Staged Image-guided Robotic Radiosurgery and Deferred Chemotherapy to Treat a Malignant Glioma During and After Pregnancy - 2018	Romanelli P, Paiano M, Crocamo V et al - Cureus	Case report	1 (pregnant woman)	Aim was growth control and the safe delivery of a healthy child	Staged radiosurgery and deferred chemotherapy proved to be a safe and effective treatment to allow the delivery of a healthy child and the long-term control of an aggressive brain glioma
Reirradiation of gliomas under stereotactic conditions: Prognostic factors for survival without relapse or side effects, a retrospective study at Tours regional university hospital (France) - 2017	Lévy S, Chapet S, Scher N et al. - Cancer Radiother.	Retrospective data analysis	13	Search for factors correlated with relapse-free survival following stereotactic reirradiation in patients with recurrent glioma following radiochemotherapy and evaluate tolerance to this treatment<br>primary endpoint: Median ST	-Median ST after stereotactic radiotherapy: 14 months<br>-Survival without relapse: 3.7 months<br><br>Factors significantly influencing duration of relapse-free survival were:<br>-age (P=0.04)<br>-total dose (P=0.02)<br>-dose per fraction (P=0.04)<br>-number of fractions (P=0.01)
Radiosurgery reirradiation for high-grade	Pinzi V, Orsi C, Marchetti	Retrospective data analysis	128 (r-HGG)	Evaluate the effectiveness of salvage SRS<br>primary endpoint: Median ST	-Median ST from initial diagnosis: 32 months

glioma recurrence: a retrospective analysis - 2015	M et al. - Neurol Sci.			secondary endpoint: Toxicity	-Median ST following SRS: 11.5 months<br><br>-No patient with high-grade toxicity<br>- 7 patients with Radiation necrosis
Improvement in treatment results of glioblastoma over the last three decades and beneficial factors - 2015	Kawano H, Hirano H, Yonezawa H et al. - Br J Neurosurgery	Retrospective data analysis	223	Elucidate the trend of glioblastoma outcome and scrutinize the factors contributing to better outcome over three decades<br>primary endpoint: Median ST	-Median ST: 13.6 months<br>-Median STs for 40-Gy EBRT plus CK-SRT: 19.1 months<br>-Median STs for 60-Gy EBRT: 10.7 months
Phase II study of bevacizumab, temozolomide, and hypofractionated stereotactic radiotherapy for newly diagnosed glioblastoma - 2014	Omuro A, Beal K, Gutin Pet al. - Clin Cancer Res.	Phase II trial	40	Evaluate the effectiveness of utilizing a novel HFSRT schedule combined with temozolomide and bevacizumab in newly diagnosed glioblastoma<br>primary endpoint: OS<br>secondary endpoint: PFS	-1-year OS: 93%<br>-Median OS: 19 months<br>-Median PFS: 10 months
Increased survival using delayed gamma knife radiosurgery for recurrent high-grade glioma: a feasibility study - 2014	Dodoo E., Huffmann B., Inti Peredo et al. - World Neurosurgery	Retrospective data analysis	55 (with WHO IV tumors)	To see if there is a survival benefit for high-grade glioma recurrences when GKRS was administered after standard therapy<br>primary endpoint: OS	-Median OS: 24.5 months<br>-2-year: 51.4%<br><br>After the recurrence was treated with GKRS:<br>-Median OS:11.3 months -2-year survival: 22.9%
Cyberknife stereotactic radiosurgery for the re-irradiation of brain lesions: a single-centre experience – 2014	Greto D, Livi L, Bonomo P, et al.G.- Radiol Med.	Retrospective data analysis	13	Evaluate the feasibility and clinical benefit of Cyberknife SRS in patients with recurrent, pre-irradiated brain lesions<br>primary endpoint: Response rates<br>secondary endpoint: side effects	Response rates:<br>-complete response: 1 case<br>-partial response: 3 cases -stable disease: 2 cases<br><br>-Local progression: 0 cases<br><br>-2 patients developed acute grade 2 toxicity requiring an increase of steroid medication
Hypofractionated stereotactic reirradiation for recurrent	Yazici G, Cengiz M, Ozyigit G et al. - J	Retrospective data analysis	37	Evaluate the results of re-irradiation of locally recurrent glioblastoma patients with an image-guided,	-Median ST following SRT: 10.6 months

glioblastoma - 2014	Neurooncol.			fractionated, frameless stereotactic radiotherapy technique<br>primary endpoint: Median ST<br>secondary endpoint: Tumor progression	-OS following initial treatment: 35.5 months<br>- Median ST of Patients with chemotherapy after SRT: 16.8 months<br>- Median ST of Patients without chemotherapy after SRT: 9.7 months
Optic nerve glioma treatment with fractionated stereotactic radiotherapy - 2013	Uslu N, Karakaya E, Dizman A et al.- Neurosurg Pediatr.	Case report	1 (11 years old)	Show effect of FSRT via the CyberKnife on visual acuity and local control in a child with an ONG	-Time to progression following SRT: 7.9 months<br>-No treatment-related side effects were observed<br>-A significantly decreased tumor size were observed
Hypofractionated stereotactic radiotherapy for unifocal and multifocal recurrence of malignant gliomas - 2013	McKenzie JT, Guarnaschelli JN, Vagal AS et al.- J Neurooncol.	Retrospective data analysis	35	Evaluate the efficacy and safety of SRT for unifocal and multifocal recurrence of malignant gliomas<br>primary endpoint: OS	-Median OS for patients treated for multifocal recurrence: 7.9 months<br>-Median OS for patients with unifocal recurrence: 10 months<br>-Local control of the SRT treated lesion(s) 6 months after SRT was associated with a significant improvement in survival ($p \leq 0.01$)
A volumetric study of CyberKnife hypofractionated stereotactic radiotherapy as salvage for progressive malignant brain tumors: initial experience - 2013	Cole A. Giller, Brian D et al. - Neurological Research	Retrospective data analysis	18	Evaluate if hypofractionated CyberKnife radiotherapy is a good alternative when single-shot radiosurgery is no option in the thearpy<br>primary endpoint: Tumorsize	-Thirteen of the 18 tumors (72%) showed a volume decrease<br>-Average volume change was a decrease of 16 ± 58%
Phase II trial of radiosurgery to magnetic resonance spectroscopy-defined high-risk tumor volumes in patients with	Einstein DB, Wessels B, Bangert B et al.- Int J Radiat Oncol Biol Phys.	Phase II trial	35	Determine the efficacy of a Gamma Knife SRS boost to areas of high risk determined by MRS functional imaging in addition to standard radiotherapy for patients with GBM<br>primary endpoint: Median ST<br>secondary endpoint: Toxicity	-Median ST: 15.8 months<br>-Median ST RPA Class 4: 18.7 months<br>-Median ST RPA Class 5: 12.5 months<br>-Median ST RPA Class 6: 3.9 months

glioblastoma multiforme - 2012					-Grade 3/4 toxicities possibly attributable to treatment: 11%
Efficacy and toxicity of CyberKnife re-irradiation and "dose dense" temozolomide for recurrent gliomas - 2012	Conti, A., Pontoriero, A., Arpa, D. et al - Acta Neurochir. 154	Non-randomised controlled study	23 (12 combined TMZ and SRS)	Evaluate the efficacy and toxicity of CyberKnife SRS alone and combined with a "dose-dense" administration of TMZ for recurrent GBM<br>primary endpoint: Median ST	-Median survival for:<br><br>patients who underwent SRS/TMZ: 12 months<br>patients who received SRS alone: 7 months<br><br>6-month progression-free survival of the patients who underwent SRS/TMZ was 66.7%; for patients who underwent SRS alone it was 18%
Temozolomide versus standard 6-week radiotherapy versus hypofractionated radiotherapy in patients older than 60 years with glioblastoma: the Nordic randomised, phase 3 trial - 2012	Malmström A, Grønberg BH, Marosi C et al.- Lancet Oncol.	Phase 3 trial, Randomised controlled study	342	Asses the optimum palliative treatment in patients aged 60 years and older with glioblastoma<br>primary endpoint: OS	-Median OS with temozolomide: 8.3 months<br>-Median OS with standard radiotherapy: 6 months<br>-Median OS with hypofractionated radiotherapy: 7.5 months
1H-MR spectroscopy guided gamma knife radiosurgery for treatment of glioma - 2012	Shen G, Xu L, Xu M, Geng M et al.- Turk Neurosurg.	Randomized controlled trial	20 (randomly divided into MRI group and MRI plus MRS group)	To observe the outcomes of 1H- MR-spectroscopy guided gamma knife surgery for treatment of glioma	-65% patients were successfully treated, of whom 6 were in the MRI group and 7 in the MRI plus MRS group<br><br>-50% patients suffered from cerebral edema during treatment, including 8 in the MRI group and 2 in the MRI plus MRS group
Increased migration of a human glioma cell line after in vitro CyberKnife irradiation - 2011	Canazza A, Chiara Calatozzolo L, Luisa, Achille et al.- Cancer Biology & Therapy	Preclinical research (U87 cell cultures)	-	Analyze the in vitro effects of irradiation on the migration of glioma cells; Spheroids of glioma cells were treated either by CIR or by CK-driven intermittent irradiation and migration of glioma cells from the spheroids invading the matrix was assessed for the following days	Treatment with CK-driven IIR significantly increased the migration ability of spheroid-derived glioblastoma cells<br><br>Treatment with CIR did not induce any significant change in invasion ability<br><br>β1-integrin was downregulated in glioblastoma cells after treatment with CIR,

					while the opposite was found after exposure to CK-driven IIR<br>-Median ST from re-irradiation: 11 months
Treatment of recurrent glioblastoma with stereotactic radiotherapy: long-term results of a mono-institutional trial – 2011	Maranzano E, Anselmo P, Casale M, et al.C - Tumori.	Retrospective data analysis; mono-institutional trial	22	Evaluate long-term outcome of 22 recurrent glioblastoma patients re-irradiated with radiosurgery or fractionated stereotactic radiotherapy<br>primary endpoint: Median ST<br>secondary endpoint: side effects	- After re-irradiation, 1 lesion was in partial remission, 16 lesions were stable, and the remaining 7 were in progression.<br><br>-23% of the patients submitted to radiosurgery developed asymptomatic brain radionecrosis
In vitro effects of CyberKnife driven intermittent irradiation on glioblastoma cell lines - 2011	Canazza A, De Grazia U, Fumagalli L et al.-Neurol Sci.	Preclinical research (A172 & U87 cell cultures)	-	Compare intermittent irradiation by using a CyberKnife with continuous irradiation by using a conventional linear accelerator	In both glioma cell lines, treatment with IIR with CK resulted in a significant decrease in clonogenic survival, if compared with the survival at the same total dose delivered by CIR
Single-arm phase II study of conformal radiation therapy and temozolomide plus fractionated stereotactic conformal boost in high-grade gliomas: final report - 2010	Balducci M, Apicella G, Manfrida S, et al. - Strahlentherapie Onkol.	Single-arm phase II study	41 (36 GBM, 5 AA)	Assess survival, local control and toxicity using FSCRT boost and temozolomide in HGGs<br>primary endpoint: Median OS<br>secondary endpoint: PFS	-Median OS: 30 months<br>-2-year survival rate: 63%<br>-Median PFS: 11 months
CyberKnife enhanced conventionally fractionated chemoradiation for high grade glioma in close proximity to critical structures - 2010	Oermann E, Collins BT, Erickson KT et al. - J Hematol Oncol.	Non-randomised controlled study	24 (12 GBM, 12 AG)	Investigate the safety and efficacy of utilizing highly conformal and precise CyberKnife radiotherapy to enhance conventional radiotherapy in the treatment of high grade glioma<br>primary endpoint: Median ST<br>secondary endpoint: Tumor progression	-Median survival of the GBM patients:18 months<br>-2-year survival rate: 37%<br>-Median survival of the AG patients:not reached<br>-4-year survival rate: 71%<br><br>-75% of the GBM patients had a local progression<br>-Median time to local progression: 16 months<br>-50 % of the AG patients had a local progression<br>-Median time to local progression: 33 months

Hypofractionated stereotactic radiation therapy: an effective therapy for recurrent high-grade gliomas - 2010	Fogh SE, Andrews DW, Glass J, et al.. – J Clin Oncol	Retrospective data analysis	147	Determine the efficacy and toxicity profile of H-SRT alone or in addition to repeat craniotomy or concomitant chemotherapy	Improvement in survival from H-SRT:<br>-Younger age (P = .001)<br>-Smaller GTV (P = .025)<br>-Shorter time between diagnosis and recurrence (P = .034)<br><br>No significant benefit of surgical resection or chemotherapy in this population when analysis was controlled for other prognostic factors
Survival following stereotactic radiosurgery for newly diagnosed and recurrent glioblastoma multiforme: a multicenter experience - 2009	Villavicencio AT, Burneikiene S, Romanelli P, et al. - Neurosurg Rev.	Retrospective data analysis Multicenter Study	56	Review the ability of CyberKnife radiosurgery to provide local tumor control of newly diagnosed or recurrent GBM<br>primary endpoint: Median ST	-Median ST from diagnosis for the patients treated with CyberKnife as an initial therapy: 11.5 months<br>-Median ST from diagnosis for the patients treated with CyberKnife at the time of tumor recurrence/progression: 21 months<br>-Median ST from the CyberKnife treatment: 9.5 months<br>-No survival advantage in using CyberKnife in initial management of GBM patients
Clinical experience with radiation therapy in the management of neurofibromatosis-associated central nervous system tumors. - 2009	Wentworth S, Pinn M, Bourland JD, et al.- Int J Radiat Oncol Biol Phys.	Retrospective data analysis	18 (acoustic neuroma (16%), ependymo ma (6%), low-grade glioma (11%), meningio ma (60%), and schwanom ma/neurofi broma (7%))	Define the efficacy of RT in patients with neurofibromatosis-associated central nervous system tumors<br>primary endpoint: OS<br>secondary endpoint: PFS	-OS rate at 5 years: 94%<br>-Five-year PFS rate (low grade Glioma): 75%
Radiographic and histopathologic observations after combined EGFR inhibition and hypofractionated stereotactic radiosurgery in patients with recurrent	Schwer AL, Kavanagh BD, McCammon R et al.- Int J Radiat Oncol Biol Phys.	Prospective Phase I trial	15	Describe the radiographic and histopathologic changes after SRS and epidermal growth factor receptor inhibition in patients with recurrent malignant gliomas<br>primary endpoint: T1C and T2 signal volumes<br>secondary endpoint: OS	-Median pretreatmentT1C volume: 40.9cm3<br>-Median post-SRS percentage increase in T1C volume at 5-7 months: 99.6%<br>-Median T1C percentage change continued to increase to 203.0%, 228.3%, and 241.5% at 8–10, 12–13, and 15 months<br>-Median pretreatment T2 volume: 169.1cm3

malignant gliomas - 2009					-Median post-SRS percentage increase in T2 volume at 5-7 months: 37.8%<br>-Median T2 percentage increase began to show a trend back downward at 8–10, 12–13, and 15 months with corresponding values of 82.2%, 76.2%, and 56.1%
Survival following CyberKnife radiosurgery and hypofractionated radiotherapy for newly diagnosed glioblastoma multiforme - 2008	Lipani JD, Jackson PS, Soltys SG, et al.. - Technol Cancer Res Treat.	Retrospective data analysis	20	Determine whether a hypofractionated dosing schedule using CyberKnife is at least as effective as multifractionated focal external beam radiation therapy<br>primary endpoint: Median OS	-Median OS: 10 months<br>-Median OS:16 months -55% of patients alive at 12 months<br>-34% of patients alive at 24 months<br><br>Using CyberKnife and hypofractionated radiotherapy compared favorably to historic data using focal EBRT in newly diagnosed post surgical GBM patients
A phase I dose-escalation study of fractionated stereotactic radiosurgery in combination with gefitinib in patients with recurrent malignant gliomas - 2008	Schwer AL, Damek DM, Kavanagh BD et al.- Int J Radiat Oncol Biol Phys.	Phase I clinical trial	15 (11 GBM; 4 AA)	Determine the maximum tolerated dose of fractionated SRS with gefitinib in patients with recurrent malignant gliomas<br>primary endpoint: MTD<br>secondary endpoint: OS; PFS	-No patient experienced a DLT<br>-SRS dose was escalated from 18 to 36 Gy<br>-Median OS: 10 months<br>-Median PFS: 7 months
Prospective trial of gross-total resection with Gliadel wafers followed by early postoperative Gamma Knife radiosurgery and conformal fractionated radiotherapy as the initial treatment for patients with radiographically suspected, newly	Smith KA, Ashby LS, Gonzalez LF, Brachman DG et al.- J Neurosurg.	Phase I/II prospective clinical trial	27	Determine whether increased local control & improved survival can be achieved in patients with GBM who undergo aggressive resection, Gliadel wafer implantation, GKS, and fractionated radiotherapy as the initial treatment<br>primary endpoint: Median ST<br>secondary endpoint: local tumor control	-Median ST: 50 weeks<br>-2-year survival rate: 22%<br><br>-2 patients remain alive at the time of the report with no clinical or radiographic evidence of disease at > 189 and 239 weeks posttreatment<br><br>-Significant difference in survival between patients whose tumors contained the methylated and unmethylated MGMT promoter, 103 versus 45 weeks<br><br>- Local tumor control was achieved in 53% of patients

diagnosed glioblastoma multiforme - 2008					
Efficacy of stereotactic radiosurgery as a salvage treatment for recurrent malignant gliomas - 2008	Kong DS, Lee JI, Park K et al..- Cancer	Prospective cohort study	114	Determine the efficacy of SRS as a salvage treatment in patients with recurrent malignant gliomas<br>primary endpoint: OS<br>secondary endpoint: PFS	-Median OS from the time of diagnosis: 37.5 months (grade 3 gliomas), 23 months (glioblastomas)<br><br>-Median PFS after SRS: 8.6 months (grade 3 gliomas), 4.6 months (glioblastomas)<br><br>-Compared with historic control group, SRS significantly prolonged survival in patients with recurrent glioblastomas but not in patients with recurrent grade 3 gliomas
Fractionated stereotactic radiotherapy boost and weekly paclitaxel in malignant gliomas clinical and pharmacokinetics results - 2007	Ashamalla H, Zaki B, Mokhtar B et al. - Technol Cancer Res Treat.	Prospective clinical trial	25 (23 GBM; 2 AA)	Study the role of adding weekly Paclitaxel to Fractionated Stereotactic Radiation Therapy (FSRT) in the treatment of Malignant Gliomas<br>primary endpoint: OS	-OS: 14 months<br>-Use of weekly Paclitaxel and FSRT in Gliomas is well tolerated with a survival of 14 months<br>-Improvement of survival of RPA classes IV, V, VI
Fractionated stereotactic radiotherapy using gamma unit after hyperbaric oxygenation on recurrent high-grade gliomas - 2007	Kohshi K, Yamamoto H, Nakahara A et al.- J Neurooncol.	Retrospective data analysis	25 (11 GBM; 14 AA)	Reduce this complication and enhance the radiation effect to hypoxic cells of high-grade gliomas, they performed noninvasive FSRT using a Gamma unit combined with HBO therapy for the treatment of recurrent disease<br>primary endpoint: Median ST	-Median ST AA: 19 months<br>-Median ST GBM: 11 months
A phase II trial of accelerated radiotherapy using weekly stereotactic conformal boost for supratentorial glioblastoma multiforme: RTOG 0023 - 2006	Cardinale R, Won M, Choucair A et al.- Int J Radiat Oncol Biol Phys.	multi-institutional phase I trial	76	Assess the feasibility, toxicity, and efficacy of dose-intense accelerated radiation therapy using weekly FSRT boost for patients with GBM<br>primary endpoint: Median ST<br>secondary endpoint: Toxicity	-Median ST: 12.5 months<br><br>Toxicity included:<br>-3 Grade 4 chemotherapy<br>-3 acute Grade 4 radiotherapy<br>-1 Grade 3 late
CyberKnife stereotactic radiotherapy for patients with	Yoshikawa K, Saito K, Kajiwara K et al.-	Retrospective data analysis	25 (18 GBM; 7 AA)	Quantify the use of the CyberKnife in the treatment of malignant glioma<br>primary endpoint: Median ST	-Median ST after diagnosis in GBM patients: 20.7 months<br>- Of 7 AA patients, 6 were alive at the time of analysis

malignant glioma - 2006	Minim Invasive Neurosurg.				(follow-up periods ranging from 11.4 to 52.8 months)<br><br>-Median ST in Patients younger than 70 years: 37.1 months<br>-Median ST in Patients older than 70 years: 12.4 months
Survival and quality of life after hypofractionated stereotactic radiotherapy for recurrent malignant Glioma - 2006	Ernst-Stecken A, Ganslandt O, Lambrecht U et al. - J Neurooncol	Prospective clinical trial	15	Evaluate efficacy and quality of life in patients with recurrent malignant glioma after hypofractionated stereotactic radiotherapy<br>primary endpoint: PFS<br>secondary endpoint: Tumor progression	PFS:<br>-at 6 months: 75%<br>-at 12 months: 53%<br><br>Rate of … (after a median follow-up of 9 months)<br>-remission: 27%<br>-no change in tumor size: 33%<br>-progressive disease: 40%
Reirradiation of recurrent WHO grade III astrocytomas using fractionated stereotactic radiotherapy (FSRT). - 2005	Combs SE, Gutwein S, Thilmann C et al.. - Strahlenther Onkol.	Retrospective data analysis	40	Assess the effect of reirradiation in recurrent WHO grade III astrocytomas<br>primary endpoint: Median OS and PFS<br>secondary endpoint: Toxicities	-Median OS: 48 months<br>-5-year OS rate: 49.5%<br>-10-year OS rate: 24.7%<br>-Median PFS from time of reirradiation: 8 months<br><br>-No toxicities > CTC grade 2 developed<br>-Median OS: 16.2 months
Radiosurgery in the treatment of malignant gliomas: the University of Florida experience - 2005	Ulm AJ 3rd, Friedman WA, Bradshaw P et al.- Neurosurgery.	Retrospective data analysis	100	Review a single-institution's 12-year experience of treating malignant gliomas with linear accelerator-based radiosurgery<br>primary endpoint: OS	-Compared with historical controls, this cohort of patients demonstrated a decreased survival for RPA Class I and II patients, similar survival for Class III and IV patients, and increased median survival for Class V patients
Stereotactic radiosurgery versus fractionated stereotactic radiotherapy boost for patients with glioblastoma multiforme – 2004	Cho KH, Hall WA, Lo SS, Dusenbery KE. - Technol Cancer Res Treat.	Retrospective data analysis	24	Aim of this study is to evaluate the efficacy of SRB in patients with GBM by comparing two different regimens, single dose or fractionated treatment<br>primary endpoint: Median ST	-2-year survival rate following SRB: 34%<br>-Median survival time: 16 months<br>-No difference in survival between SRS and FSRT treated patients<br>-Potentially less late complications associated with FSRT
Radiation necrosis and brain edema association with	Sato K, Baba Y, Inoue M et al.. - Acta	Retrospective data analysis	61	Analyze frequency and risk factors of radiation necrosis after CyberKnife treatment	-4 patients showed symptomatic radiation necrosis

CyberKnife treatment - 2003	Neurochir Suppl.				-2 patients required necrotomy through craniotomy<br><br>Risk factors:<br>-Inhomogeneity of the maximum dose area<br>-Number of fractions and dose per fraction
Phase II study of high central dose Gamma Knife radiosurgery and marimastat in patients with recurrent malignant glioma - 2002	Larson DA, Prados M, Lamborn KR et al.- International Journal of Radiation Oncology *Biology*Physics	Prospective Phase II study	26	Assess the outcome of high central dose Gamma Knife radiosurgery plus marimastat in patients with recurrent malignant glioma<br>primary endpoint: PFS<br>secondary endpoint: OS	-Median PFS after radiosurgery for Grade 3 and 4 patients: 31 and 15 weeks<br>-Median OS time after radiosurgery: 68 and 38 weeks<br>-Median survival time after radiosurgery in the historical patients: 59 and 44 weeks
Gamma knife radiosurgery for pilocytic astrocytomas - 2002	Boëthius J, Ulfarsson E, Rähn T, et al. - J Neurosurg.	Retrospective data analysis	19	Evaluate the efficacy of gamma knife radiosurgery for pilocytic astrocytoma<br>primary endpoint: Tumor control<br>secondary endpoint: Side effects	-Tumor control was achieved in 18 patients (either a diminished volume, which stabilized into a radiologically demonstrated residuum, or a total disappearance)<br>-1 patient underwent surgery for radionecrosis (undergone a course of conventional radiotherapy prior to GKS)<br>-1 patient developed a radiation-induced hemiparesis<br>-2 patients developed tumor cysts after the GKS
Preliminary report of a phase I study of combined fractionated stereotactic radiosurgery and conventional external beam radiation therapy for unfavorable gliomas - 2002	Regine WF, Patchell RA, Strottmann JM et al. – 2000	Retrospective data analysis	14	Determine the tolerance and toxicities of FSRS given in combination with CEBRT<br>primary endpoint: Side effects<br>secondary endpoint: OS	-8 patients underwent reoperation for signs, symptoms, or radiologic imaging consistent with either radionecrosis and/or tumor progression<br>-3 patients had necrosis without evidence of glial neoplasm on pathologic exam<br>-5 patients had reversible grade 3 toxicity who had reversal of pre-reoperation progressive neurologic signs and/or symptoms<br><br>-7 patients died of disease progression at 9–15 months

Combined stereotactic split-course fractionated gamma knife radiosurgery and conventional radiation therapy for unfavorable gliomas: a phase I study - 2000	Regine WF, Patchell RA, Strottmann JM et al.- Journal of Neurosurgery	Phase I study	18 (11 patients with malignant gliomas, six with low-grade gliomas, and one with a recurrent glioma)	Determine the tolerance and toxicities of split-course fractionated gamma knife radiosurgery given in combination with conventional external-beam radiation therapy<br>primary endpoint: OS<br>secondary endpoints: side effects and tumor size	-7 patients were alive at 22–51 months<br>-6 patients remained alive for 3 to 60 months<br><br>-38% of the patients had necrosis without evidence of tumor<br><br>-2 had a partial (> or = 50%) reduction in volume and nine had a minor (> 20%) reduction in size
Accelerated radiotherapy regimen for malignant gliomas using stereotactic concomitant boosts for dose escalation - 1998	Cardinale RM, Schmidt-Ullrich RK, Benedict SH, Zwicker RD, Han DC et al.- Radiat Oncol Investig.	Phase I study	12 (9 GBM; 3 AA)	Determine the feasibility and toxicities of an accelerated treatment program by using a concomitant stereotactic radiotherapy boost given weekly during a course of standard EBXRT in patients with malignant gliomas<br>primary endpoint: Median ST<br>secondary endpoints: side effects	-Median ST GBM: 16 months<br>-Median ST AA: 33 months<br><br>-Radionecrosis occurred in 4 patients
Radiosurgery and accelerated radiotherapy for patients with glioblastoma - 1997	Shenouda G, Souhami L, Podgorsak EB et al.- Can J Neurol Sci.	Phase I study	14	Assess the feasibility, toxicity, and local control of SRS followed by AEBR for patients with glioblastoma multiforme<br>primary endpoint: Median ST	-Median ST: 40 weeks<br>-Survival rate at 12 months: 43%<br>-Survival rate at 18 months: 14%
Gamma Knife radiosurgery for the treatment of brainstem tumors - 1995	Hirato M, Nakamura M, Inoue HK, Ohye C et al.- Stereotact Funct Neurosurg.	Retrospective data analysis	10 (2 with gliomas)	Evaluate if GammaKnife Radiosurgery reduces the tumor size more than conventional radiation therapy<br>primary endpoint: Tumor size	In the 2 cases with glioma, the tumor remained unchanged or continued to grow
Stereotactic radiosurgery for recurrent gliomas -1994	Chamberlain MC, Barba D, Kormanik P et al..- Cancer	Clinical Trial, Phase II	20	Evaluate the efficacy of single fraction, high dose stereotactic radiotherapy or radiosurgery in patients with gliomas<br>primary endpoint: OS<br>secondary endpoint: Tumor progression	-19 patients with tumor progression<br>-median time-to-tumor progression: 9 months<br><br>-14 patients died from tumor progressive (median survival: 7 months)<br><br>-5 patients were alive (median follow-up: 19 months)

Stereotactic radiosurgery for glioblastoma multiforme: report of a prospective study evaluating prognostic factors and analyzing long-term survival advantage - 1994	Mehta MP, Masciopinto J, Rozental J, Levin A et al.-Int J Radiat Oncol Biol Phys.	Prospective trial	31	Evaluation of the toxicity and efficacy of radiosurgery with external beam radiotherapy in the management of newly diagnosed glioblastoma<br>primary endpoint: Median ST<br>secondary endpoint: Side effects	-Median survival: 42 weeks<br>-12 months survival rate: 38%<br>-24 months survival rate: 28%<br><br>-13% of the patients developed clinically significant necrosis<br>-No significant acute toxicity was encountered
Survival comparison of radiosurgery-eligible and -ineligible malignant glioma patients treated with hyperfractionated radiation therapy and carmustine: a report of Radiation Therapy Oncology Group 83-02 - 1993	Curran WJ Jr, Scott CB, Weinstein AS, et al.J Clin Oncol.	Randomized phase I/II trial	778	Identify the malignant glioma patients treated in a trial of hyperfractionated radiotherapy and carmustine who may have been eligible for a SRS boost; and compare survival of such patients with that of those considered SRS-ineligible	-SRS-eligible patients enrolled on RTOG 83-02 had survival superior to that of the SRS-ineligible group -> this advantage is mainly due to the selection of a subgroup with a high minimum KPS
